# Coproducing a library of videos to support families caring for children with gastrostomies: A mixed‐methods evaluation with family carers and clinicians

**DOI:** 10.1111/hex.13449

**Published:** 2022-02-09

**Authors:** Bethan Page, Alex C. H. Lee, Emily J. Harrop, Tania Beale, Alison Sharrard, Nick Yeung, Charles A. Vincent

**Affiliations:** ^1^ Department of Experimental Psychology University of Oxford Oxford UK; ^2^ Department of Paediatrics Oxford University Hospitals NHS Foundation Trust Oxford UK; ^3^ Helen and Douglas House Oxford UK

**Keywords:** coproduction, education, family carers, gastrostomy care, multidisciplinary

## Abstract

**Introduction:**

Many families now perform specialist medical procedures at home. Families need appropriate training and support to do this. The aim of this study was to evaluate a library of videos, coproduced with parents and healthcare professionals, to support and educate families caring for a child with a gastrostomy.

**Methods:**

A mixed‐methods online survey evaluating the videos was completed by 43 family carers who care for children with gastrostomies and 33 healthcare professionals (community‐based nurses [*n* = 16], paediatricians [*n* = 6], dieticians [*n* = 6], hospital‐based nurses [*n* = 4], paediatric surgeon [*n* = 1]) from the United Kingdom. Participants watched a sample of videos, rated statements on the videos and reflected on how the videos could be best used in practice.

**Results:**

Both family carers and healthcare professionals perceived the video library as a valuable resource for parents and strongly supported the use of videos in practice. All healthcare professionals and 98% (*n* = 42) of family carers agreed they would recommend the videos to other families. Family carers found the videos empowering and easy to follow and valued the mixture of healthcare professionals and families featured in the videos. Participants gave clear recommendations for how different video topics should fit within the existing patient pathway.

**Discussion:**

Families and healthcare professionals perceived the videos to be an extremely useful resource for parents, supporting them practically and emotionally. Similar coproduced educational materials are needed to support families who perform other medical procedures at home.

**Patient or Public Contribution:**

Two parent representatives attended the research meetings from conception of the project and were involved in the design, conduct and dissemination of the surveys. The videos themselves were coproduced with several different families.

## 1 INTRODUCTION

Historically family members have helped young children and older adults with activities of daily living, such as helping with dressing, eating and bathing, but nursing and medical tasks were once solely the domain of nurses and doctors. Over the last few decades, there has been a dramatic transformation in the type of care undertaken by family caregivers at home.^1^, ^2^ In paediatrics, there are increasing numbers of children dependent on medical technologies (e.g., feeding tubes, ventilator equipment to assist breathing) who are predominantly cared for at home by their parents.^3^, ^4^ Parents caring for these children have to learn to perform specialist nursing tasks, troubleshoot problems and in some cases, acquire sophisticated monitoring and diagnostic skills.^5^ For example, parents caring for children with gastrostomies (a feeding device inserted during surgery for delivering nutrition and medication directly into the stomach) are responsible for administering feeds and medications, cleaning and caring for the stoma site, performing maintenance tasks such as changing the water in a gastrostomy button and managing problems such as sore and leaking stoma sites and blocked tubes.^6^ Parents may also learn to change a gastrostomy button, and must be able to recognise and manage safety‐critical issues such as a dislodged gastrostomy button. This is highly skilled work.

A key challenge is how to prepare and support parents to provide this specialist care. In an analysis of incident reports for children with feeding tubes, healthcare professionals identified inadequate training for family carers as a significant cause of concern, with some children coming to a harm as a result of inadequate training for families.^7^ Our research on gastrostomy care has shown that many parents do not feel adequately prepared to provide the required care when they first go home after their child's surgery and many reported feeling very anxious. In a survey we conducted, parents recommended that videos featuring families and healthcare professionals would be a useful form of training and support, in addition to face‐to‐face training from nurses.^8^ Participants recommended topics to cover in videos and emphasized the need to provide emotional support as well as practical skill development. Understanding the experience and perspectives of parents providing this care is critical to developing appropriate and effective resources. Coproduction needs to be at the heart of developing training materials for families to ensure the resources fully meet their needs, and recognise the lived experiences and expertise of families who provide this care every day.

In this study, we report on the evaluation of a library of videos produced by our multidisciplinary team to support families caring for children with gastrostomies. The videos were developed by and feature a range of different families and healthcare professionals (e.g., hospital‐ and community‐based children's nurses, a paediatrician and a surgeon). Successful implementation of any intervention depends on the acceptability of the intervention to both intervention deliverers (i.e., healthcare professionals who support and train parents) and recipients (parents).^9^ The videos were evaluated by family carers from across the United Kingdom and a sample of healthcare professionals from the region where the videos were developed and a second region in the United Kingdom. The primary aim of the study was therefore to evaluate the acceptability of the library of videos with families and healthcare professionals who care for children with gastrostomies, including the perceived impact and benefits of the videos, and satisfaction with the content and presentation of the videos. The second aim was to explore healthcare professionals' and families' views on how to make the best use of the videos in practice.

## 2 METHODS

### 2.1 Development of the video library

The videos evaluated in this paper were produced by our team of researchers, healthcare professionals and parents. We designed the videos to support families from when their child is referred for a gastrostomy, through the immediate postoperative period and extending to long‐term care at home. The content and topic list for the videos was informed by recommendations from a survey with 146 family carers^8^ and advice from our stakeholder group, which consisted of parent representatives, children's nurses from the community, specialist nurses, paediatricians, a gastrostomy surgeon and researchers. The stakeholder group met regularly over a 3‐year period.

The videos were designed to feature families caring for their children in the home environment, as recommended by our parent representatives. Our stakeholder group agreed that teaching in the videos needed to be delivered by expert parents and a multidisciplinary team of healthcare professionals recognizing the different types of expertise and support they can offer families. The aim was that parents and clinicians would copresent where possible. The videos were purposely designed to teach parents not only how to care for their child's gastrostomy and troubleshoot common problems but also to address emotional challenges and for families to learn from the experiences of other parents as well as clinicians. Critically the videos are also intended to reassure families and reduce anxiety; the videos address common concerns and recognise that many families are scared at the start of their journey (e.g., anxious about what life will be like when their child has a feeding tube, anxious about being seen as a failure, anxious about the judgements of others). The need to reassure families and reduce anxiety was seen as particularly important by our parent representatives.

**Figure 1:**
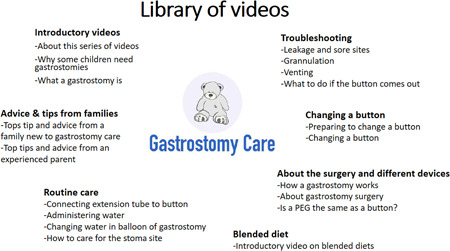
List of topics included in the library of videos for families caring for children with gastrostomies. A subset of these videos were watched by participants in the survey

Throughout the development process, the videos were informally evaluated by the stakeholder group to ensure the content of the videos was appropriate and consistent with best practice. At the time of evaluation, the video library consisted of 19 videos (see Figure 1): An additional video on differences in practice has since been added. The videos are available here (https://www.oxstar.ox.ac.uk/more/supporting-parents/watch-the-videos) and are searchable on YouTube.

### 2.2 Development and design of the survey

We designed a mixed‐methods survey to evaluate the videos, with two slightly different versions adapted for families and for healthcare professionals. Both versions had a roughly even number of qualitative and quantitative questions. The qualitative data were intended to help support, illustrate and expand the quantitative data.^10^ The surveys were drafted, piloted and revised in consultation with our multidisciplinary stakeholder group. The evaluation of the videos was approved as a service evaluation by Oxford University Hospitals NHS Foundation Trust and the University of Oxford.

### 2.3 Survey content

The surveys consisted of four sections: (i) information about the participants, (ii) watching a sample of videos, (iii) evaluating the content of the videos and (iv) using the videos in practice. Participants were shown the full list of video topics included in the library (see Figure 1) and asked to watch a representative sample of six videos that were preselected by the team to cover a range of different topics. We did not ask participants to watch all 19 videos in the library as this would be too time‐consuming. Participants were then asked to rate statements assessing the acceptability of the videos. The questions were designed to capture different components of acceptability^9^: burden (e.g., ‘the videos are an appropriate length’), experience (e.g., ‘the information in the videos is easy to understand’), perceived effectiveness (e.g., ‘the videos will help prepare parents to care for their child's gastrostomy’) and intention (‘I would recommend these videos to parents’). Participants also answered open‐ended questions on what they liked about the videos, what they found most helpful, what could be improved, what they learnt from the videos and any additional topics they wanted to see covered. The final section asked to reflect on how the videos could be best used in practice. The full survey is available in File S1.

### 2.4 Sampling and recruitment

For the family carer survey, the inclusion criteria were any parent or family carer who provides gastrostomy care to a child or young person at home or has a child on the waiting list for gastrostomy surgery. By family carer, we included any unpaid carer (parent, relative, friend) who actively participated in caring for a child or young person with a gastrostomy. To take part family carers needed to be at least 18 years old. Families who participated in our previous study^8^ and agreed to be contacted again were invited to take part in this survey (*n* = 102). All of these families had at least 1 year's experience caring for their child's gastrostomy. To recruit some families who were new to gastrostomy care, the surgical lead for our region invited some families on the waiting list for gastrostomy surgery or who had recently had surgery in the region where the study took place and where the videos were developed. Our parent representatives also contacted relevant charities and leads for closed Facebook groups to help recruit families who had recently had gastrostomy surgery. Due to the COVID‐19 pandemic, far fewer surgeries for gastrostomies have taken place compared to usual. All participants (family carers and professionals) received a £10 voucher for taking part.

For the healthcare professionals' survey, participants needed to be healthcare professionals who support children and young people who have gastrostomies (e.g., community nurses, hospital‐based children's nurses, surgical feeding teams, paediatricians, dieticians, respite and school staff, registrars and junior doctors etc.). We recruited a range of different types of healthcare professionals who support families with gastrostomies. The surgical lead for gastrostomies in the region where the resources were developed compiled a list of relevant healthcare professionals from our region (*n* = 58) who were invited by email to take part and *n* = 21 completed the survey. To explore whether the videos would be suitable for use outside of the region in which they were developed, we then contacted a second region to be involved in the evaluation. The lead clinician for this region contacted *n* = 24 healthcare professionals (those invited were asked to forward the invite if there was somebody else more suitable within their organization). In total *n* = 12 completed the survey from this second region.

### 2.5 Analysis

Participants were included if they completed the full survey defined as viewing all pages of the survey and completing at least 90% of the quantitative questions. Some of the family carer responses were excluded as probable spam responses, based on an assessment of the open‐ended responses (these occurred after one of the charities posted the link on Twitter). Descriptive statistics were computed for all close‐ended questions, using SPSS Statistics 25. Participants who did not complete the full survey were excluded. The open‐ended questions were coded in NVivo 12 using inductive content analysis, to group responses based on surface level of meaning.^11^ Each meaningful statement was coded and grouped into categories emerging from the data. These were summarized in the text and illustrated with quotes from participants. In the first section of the survey (the evaluation of the content of the videos) our analysis focuses primarily on family carers' responses, with some feedback from healthcare professionals included, since families are the primary audience for the videos. Conversely, the second section on using the videos in practice focuses primarily on the responses of healthcare professionals, with some additional feedback from families, since it is healthcare professionals who lead on implementing the videos into routine practice.

## 3 RESULTS

### 3.1 Participants

There were 344 responders who viewed the first page of the family carer survey and 69 responders for the healthcare professional version. Forty‐three family carers and 33 healthcare professionals completed the full survey. Table 1 provides an overview of the characteristics of the family carers and healthcare professionals who participated.

**Table 1:** Sample characteristics

Characteristics	*N* (%)
Family carers	43
Relationship to child	
Mother	37 (86%)
Father	5 (12%)
Other family members	1 (2%)
Age of child (years)	
0–3	12 (28%)
4–7	19 (44%)
8–11	3 (7%)
12–16	9 (21%)
Time since gastrostomy surgery	
On waiting list	2 (5%)
<1 year	5 (12%)
1–2 years	11 (26%)
3–4 years	16 (37%)
5+ years	9 (21%)
Healthcare professionals	33
Job role	
Community Children's Nurse	7 (21%)
Other community‐based nurses (e.g., respite, school)	9 (27%)
Paediatrician	6 (18%)
Dietician	6 (18%)
Hospital‐based nurse	4 (12%)
Paediatric surgeon	1 (3%)
Involvement in teaching about gastrostomy care	
Families and healthcare professionals	18 (55%)
Families only	4 (12%)
Healthcare professionals only	4 (12%)
Never been involved in gastrostomy teaching	7 (21%)
Location healthcare professional works	
Region A (where the resources were developed)	21 (64%)
Region B	12 (36%)

### 3.2 Participants' ratings of the training videos

Both family carers and healthcare professionals perceived the video library as a valuable resource for parents and strongly supported the use of the videos in practice. As Table 2 shows, nearly all statements were rated as ‘strongly agree’ or ‘agree’ by over 90% of family carers and healthcare professionals. Notably, 77% of family carers and 88% of healthcare professionals ‘strongly agreed’ that they would recommend the videos to parents, and 84% of family carers and 91% of healthcare professionals ‘strongly agreed’ that the videos would be helpful to families new to gastrostomy care (with the remaining agreeing). A small number of family carers disagreed with the statement ‘I have no concerns about the accuracy of advice given in the videos’. Their concerns related to small differences in practice across the country, for example, some families commented that button tube pads were not recommended in their area or using enplug stoppers if the button comes out: ‘Some medics are vastly against tube pads or indeed using maxitrol so it could be made clearer that opinions will vary’ [Mother, 1–2 years' experience].

**Table 2:** Ratings of statements evaluating the content of training videos by family carers

		Family carers' ratings (F)/Healthcare professionals' ratings (H)					
		Strongly agree, *N* (%)	Somewhat agree, *N* (%)	Neither agree or disagree, *N* (%)	Somewhat disagree, *N* (%)	Strongly disagree, *N* (%)	Missing, *N* (%)
The information in the videos is easy to understand	F	35 (81%)	7 (16%)	0	0	0	1 (2%)
	H	27 (82%)	6 (18%)	0	0	0	0
There is a good mixture of healthcare professionals and families in the videos	F	37 (86%)	4 (9%)	1 (2%)	1 (2%)	0	0
	H	28 (85%)	5 (15%)	0	0	0	0
I have no concerns about the accuracy of advice given in the videos (F only)	F	24 (56%)	11 (26%)	3 (7%)	2 (5%)	2 (5%)	1 (2%)
The information in the videos is consistent with best practice guidance (H only)	H	18 (55%)	12 (36%)	2 (6%)	1 (3%)	0	0
The videos are an appropriate length	F	30 (70%)	11 (26%)	1 (2%)	0	0	1 (2%)
	H	22 (67%)	9 (27%)	0	1 (3%)	0	
There is a good range of topics covered	F	34 (79%)	9 (21%)	0	0	0	0
	H	26 (79%)	6 (18%)	1 (3%)	0	0	0
The videos will help prepare parents to care for their child's gastrostomy at home	F	31 (72%)	10 (23%)	1 (2%)	1 (2%)	0	0
	H	23 (70%)	10 (30%)	0	0	0	0
The videos will help families to feel more confident	F	33 (77%)	9 (21%)	1 (2%)	0	0	0
	H	27 (82%)	5 (15%)	1 (3%)	0	0	0
The videos will be useful to families new to gastrostomy care	F	36 (84%)	7 (16%)	0	0	0	0
	H	30 (91%)	3 (9%)	0	0	0	0
The videos will be useful to families who are more experienced at caring for their child's gastrostomy	F	11 (26%)	16 (37%)	9 (21%)	4 (9%)	3 (7%)	0
	H	8 (24%)	17 (52%)	7 (21%)	1 (3%)	0	0
I would recommend these videos to parents	F	33 (77%)	9 (21%)	1 (2%)	0	0	0
	H	29 (88%)	4 (12%)	0	0	0	0
I would recommend these videos to my colleagues (H only)	H	24 (73%)	9 (27%)	0	0	0	0

### 3.3 General reflections on the video library

Participants offered many reflections when asked what they liked most about the videos. The main themes were ease of understanding, balance of parents and healthcare professionals and perceived emotional impact of the videos. File S2 summarizes the comments on individual videos in more detail.

#### 3.3.1 Ease of understanding

Most family carers and healthcare professionals commented on how easy the information was to understand, and felt the videos were short and to the point. A few participants commented on the simplicity of the language and the useful diagrams and summaries of key points. One family carer, for example, commented on how useful the three‐dimensional models were in the surgery video: ‘When we had our consultant with the surgeon he drew a few scribbles on a piece of paper to explain it—however in the video you used actual models to show it’ [Mother, <1 years' experience].

#### 3.3.2 Balance of parents and healthcare professionals

Many of the family carers and healthcare professionals commented on the mixture of families and healthcare professionals featured in the videos. For example, one family carer commented ‘I think the balance between parents/carers having first‐hand experience and also clinicians is really important and done very well’ [Father, 1–2 years' experience]. Families appreciated seeing other families in the home environment: ‘I like that parents were used in this who have experience—it's reassuring for new parents to see how normal this new normal is’ [Mother, 5+ years' experience]. Some family carers and healthcare professionals commented that they really valued the range of different healthcare professionals involved from the community and hospital. A few families from Region A (where the videos were developed) commented that they valued seeing professionals involved in their child's care in the videos: ‘Lovely to see the professionals that we know from the hospital involved as adds “comfort”’ [Mother, 3–4 years' experience].

#### 3.3.3 Perceived emotional impact of videos

Some family carers commented on the emotional impact of the videos. A sense of feeling empowered was a common theme. For example, one parent with a child on the waiting list for surgery said: ‘This was so normalising and reassuring. It helped us to imagine our lives when our daughter has her button in place. They were incredibly empowering’ [Mother, child awaiting surgery]. A few family carers commented on how reassuring they found the videos, especially the advice and tips from a new parent video: ‘I found this video really moving. It was helpful and reassuring to hear about the emotions involved for this family. It made the whole process seem a bit less daunting and more real’ [Mother, child awaiting surgery]. Some healthcare professionals also commented on the potential emotional benefits of the videos.

### 3.4 Suggested improvements to the videos

Most of the comments from family carers and healthcare professionals on what to improve were minor suggestions. Some suggestions related to the quality of the videos, for example, the sound quality, or the professionals in the videos looking a little ‘stiff’. There were some suggestions for adding further specific details, or content, or highlighting small differences in practice between what was in the videos and what a family does/or is recommended in their region. A few of the family carers suggested that there should be a broader range of families featured in the videos, for example, including fathers or children with different types of needs or different ages. Suggestions for additional topics to cover in the videos included practical issues around managing supplies and reuse of equipment, different types of devices and setting up feeds and using feeding pumps. A few families and healthcare professionals commented that videos should be created for other types of care: ‘It would be great to see similar videos for other devices, for example a tracheostomy’ [Community Children's Nurse, Region A].

### 3.5 Learning from the videos

Participants were asked whether the videos had aided their own learning. For the family carers, 5 (12%) responded ‘yes a lot’, 23 (55%) responded ‘yes a little’ and 14 (33%) said the videos had not aided their own learning. For the healthcare professionals, 5 (15%) responded ‘yes a lot’, 24 (73%) responded ‘yes a little’ and only 5 (15%) said that videos had not aided their own learning.

The two parents with a child on the waiting list for surgery found it very useful to review the videos before the gastrostomy was carried out. One of these parents commented ‘We are familiar with NG tubes but didn't know anything about PEGs/buttons, except they were some kind of surgically installed port in the stomach. We now feel we understand the subject and are in a much better position to ask relevant questions in clinic’ [Father, child awaiting surgery]. The other parent commented ‘Although I had read through the leaflets I think having the videos allows us to visualize what the different terms mean, which is so helpful for making sense of, and retaining, the information. I understand better how the surgery works, what procedures are needed to care for the button and the stoma, and I'm more comfortable with the terminology’ [Mother, child awaiting surgery].

Even some very experienced family carers described learning new things from the videos, or finding the videos a helpful reminder. For example, one experienced parent said: ‘Great idea, could really have used these 10 years ago but even now they're really useful for a reminder as parents tend not to get any updated training’ [Mother, 5+ years' experience]. Healthcare professionals too commented on new learning, for example, learning more about treatments for granulation tissue: ‘Granulation is always a difficult one to resolve so good to know the favoured 5 steps the CNS [clinical nurse specialist] suggests with confidence’ [Community‐based nurse, Region B]. A few healthcare professionals also commented on learning something from the families in the videos: ‘The testimony from the mother of a girl with gastrostomy was very insightful, especially how she explained the gastrostomy to her daughter in the form of a story’ [Paediatrician, Region A]. Many of the healthcare professionals commented on the value of the videos for training staff as well as families, especially as awareness training.

### 3.6 Using the videos in practice

This section primarily focuses on the perspective of healthcare professionals as they are responsible for the organization and delivery of services, with some data and reflections from families where relevant.

#### 3.6.1 How the videos might be integrated into the existing clinical pathway

To assess how the different topics would best fit within a family's journey, participants were presented with different topic areas for the videos and asked to rate when a particular group of videos would be most appropriate to watch: (i) ‘when referred to hospital team for a gastrostomy’, (ii) ‘around the time of surgery’, (iii) ‘in the first few weeks at home after surgery’, (iv) ‘after child has had gastrostomy for a few months or years’ or (v) ‘not sure’. Participants could select more than one time point if they felt that was appropriate. Table 3 shows which topics were rated as more appropriate to watch when. Each topic consists of 1–4 individual videos (see Figure 1).

**Table 3:** Parents' and healthcare professionals' recommendations for which videos would be most helpful for parents to watch at the different stages of their journey

	Family carers' ratings (F)/Healthcare professionals' ratings (H)					
		When referred to hospital team for a gastrostomy	Around the time of surgery	In the first few weeks at home after surgery	After child has had gastrostomy for a few months or years	Not sure
Introductory videos	F	40 (93%)**	20 (47%)*	10 (23%)	5 (12%)	1 (2%)
	H	32 (97%)**	9 (27%)	3 (9%)	0	0
About the surgery and different devices	F	36 (84%)**	21 (49%)*	5 (12%)	4 (9%)	1 (2%)
	H	30 (91%)**	18 (55%)*	4 (12%)	2 (6%)	0
Advice and tips from parents	F	18 (42%)*	29 (67%)**	26 (61%)**	8 (19%)	1 (2%)
	H	17 (52%)*	20 (61%)**	17 (52%)*	4 (12%)	1 (3%)
Routine care (e.g., how to clean site)	F	8 (19%)	30 (70%)**	29 (67%)**	8 (19%)	1 (2%)
	H	5 (15%)	29 (88%)**	22 (67%)**	5 (15%)	0
Troubleshooting (e.g., over‐granulation)	F	7 (16%)	13 (30%)*	35 (81%)**	17 (40%)*	1 (2%)
	H	2 (6%)	19 (58%)*	31 (94%)**	13 (39%)*	0
Changing a button	F	8 (19%)	14 (33%)*	28 (65%)**	22 (51%)*	1 (2%)
	H	4 (12%)	15 (46%)*	26 (79%)**	14 (42%)*	0
Blended diet	F	24 (56%)*	11 (26%)	19 (44%)*	22 (51%)*	6 (14%)
	H	9 (27%)	8 (24%)	15 (46%)*	23 (70%)**	5 (15%)

*Note*: The table shows the number and percentage of participants who rated a particular topic.

* More than 30% but less than 60% of participants rated this time point as the most appropriate point in the family's journey to watch the videos.

** More than 60% of participants rated this time point as the most appropriate point in the family's journey to watch the videos.

Family carers and healthcare professionals broadly agreed on which videos would be most appropriate to watch when. The only notable disagreement was for the blended diet video. The majority of healthcare professionals recommended the blended diet video should be watched later in the pathway: 70% recommended it should be watched a few months or years after the surgery and only 27% recommended it should be watched before surgery. Conversely, 56% of families recommended that the blended diet video should be watched before surgery. Some families recommended that families ought to be aware that blended diet is an option before the surgery: ‘Blended diet can be a reassurance that food doesn't have to be off the menu forever for their child and actually a bit of normality that can make mealtimes a family affair again’ [Mother, 5+ years' experience].

The majority of family carers and healthcare professionals felt that the videos were best watched spaced out and that watching them all before the surgery would be information overload. A few parents however did indicate that they would want to watch most of the videos before their child's surgery: ‘I guess it depends on how the parents feel I personally would have found these videos helpful before operation but guess some parents may find them overwhelming’ [Mother, 3–4 years' experience]. Some healthcare professionals had clear ideas about how different topics should fit within the patient pathway, for example, commenting that they would want to send some of the videos to families before the first appointment to discuss a child's gastrostomy. A few parents and healthcare professionals also commented that the videos could help parents make the decision about whether to consent to gastrostomy surgery for their child: ‘The videos would be helpful for parents to get an in depth understanding of the operation and will really help with gaining informed consent’ [Community Children's Nurse, Region A].

#### 3.6.2 Potential dangers and risks

Participants were asked to comment on any potential dangers or risks. The majority of respondents indicated that they did not feel there were any particular dangers or risks: ‘I think any positives of new information beforehand to prepare them for their journey will outweigh any risks. With any situation there are always dangers when you introduce new procedures etc. but if families are well prepared with information this will help to reduce issues later on’ [Dietician, Region A]. Several healthcare professionals commented on the importance of using the videos alongside face‐to‐face training, and not to replace it, or assume that if a parent has watched the video they have understood it all. A few healthcare professionals and families mentioned the risk that families might watch the videos and not also seek out training: ‘Is there a risk people may watch a video rather than “bother” a professional before being trained, rather than as an aide memoire?’ [Mother, 3–4 years' experience]. There were a few comments about differences in practice or processes across the country that could cause some minor confusion.

## 4 DISCUSSION

This evaluation study demonstrates that the library of videos is highly valued by both families and healthcare professionals. Participants felt the information was clear and easy to understand, and appreciated the collaboration between families and healthcare professionals in the videos and the perceived emotional benefits of the videos. Nearly all participants agreed the videos would help families to feel confident and would help prepare families to care for their child's gastrostomy at home (measure of perceived effectiveness of the intervention). Over 90% of family carers and healthcare professionals would recommend the videos to other parents (measure of intention) and over 90% agreed that the videos were an appropriate length (minimal burden of the intervention), covered a good range of topics and featured a good mix of healthcare professionals and families. The videos were also rated by a majority as also useful for more experienced families, either as a refresher or to help with specific troubleshooting issues as needed. The evaluation has provided a clear indication of which videos might be most appropriate to watch at different time points in families' journeys, and how they might be used in practice. Overall families and healthcare professionals perceived the videos as a useful adjunct to the existing training and information provided to families caring for a child with a gastrostomy.

Many of the families in the evaluation commented that they found the videos empowering and reassuring. Families liked that the videos featured real families caring for their children at home and many found this normalizing. The videos can help families imagine what life with a child with a gastrostomy will be like. Healthcare professionals and families agreed the videos help prepare families to care for their child's gastrostomy, and can increase their confidence before they take their child home after the surgery. Our past research shows that many families report feeling very anxious in the first few weeks of caring for their child at home.^8^ Having a gastrostomy fitted is often a major life‐changing decision for families,^12^ which is recognised in the testimonies of the families in our videos. The resources provide a critical role beyond education, helping families to feel emotionally prepared and supported. It is important to remember that the video library is there to empower parents and supplement existing training and support, and should not be used as a replacement for face‐to‐face training.

Other studies have recognised the need to provide more training and information to families as a means of reducing the number of avoidable visits to the emergency department for common problems, such as problems with the stoma site or gastrostomy device,^13^ and as a means of safely reducing the length of stay in hospital for the surgery, which is not possible without more training and support for families before the hospital admission.^14^ As advocated in the NHS long‐term plan, these educational videos can help reduce pressure on hospital and community services by empowering and better‐educating families so children experience fewer complications and parents are more confident providing high‐quality routine care and troubleshooting common problems. Using videos and online resources (digitally enabled care) to support families is also more equitable and accessible than relying solely on face‐to‐face training and support, for example, subtitles can be added in other languages and the videos can be paused and rewatched as needed. There are many potential benefits of these videos for families and health services.

There is an emerging recognition of the need to provide better training to family caregivers who are performing medical procedures and technical care.^15^, ^16^ Educational videos for family caregivers for a range of different medical procedures beyond gastrostomy care are needed. We recommend that teams seeking to develop educational videos like ours for other types of medical care (e.g., stoma care, other forms of artificial feeding) work with families from the conception of the project. Coproduction in this context requires sharing power with the families, and ensuring the content of the videos are codesigned and copresented by families and healthcare professionals.^17^, ^18^ The data presented in this paper shows families valued seeing videos filmed in the home environment, rather than filming solely in clinical environments. A key strength of our resources is they recognise the expertise of family carers and the range of different types of clinicians from hospital and community services. Many family carers develop substantial expertise in their child's needs over time,^5^, ^19^ and this expertise is highly valued by inexperienced families, alongside the expertise of specialist healthcare professionals.

### 4.1 Strengths and limitations

One strength of the sample of family carers in our evaluation is that it included some families relatively new to gastrostomy care and some who were more experienced. More experienced families are better able to reflect on what is needed at the different points in their journey; however, it was also important to capture whether the videos were acceptable to families at the start of their learning journeys. The sample also included a variety of different types of healthcare professionals who support children with gastrostomies. The sample of healthcare professionals included some from outside the region in which the videos were developed; data from these participants suggests that the information in the videos is broadly consistent with guidelines in different regions of the country but there are some small differences. Since the evaluation we have added a video to the library to explicitly discuss differences in practice with families to make them aware of some differences that exist and to advise them to discuss any differences or concerns with the professionals supporting them (e.g., whether to use cooled boiled water or sterile water in the gastrostomy balloon).

The team who developed the videos conducted the evaluation, and it was not therefore an independent evaluation. The evaluation was primarily conducted by the first author (B. P.) (a researcher leading a programme of work on support for parents of children with medical complexity) who co‐ordinated the development of the videos. The evaluation surveys were developed with support from the team of clinicians and parent representatives involved in the development of the videos, which ensured the questions reflected the intended aims and benefits of the videos, and any concerns the team had about implementation. The surveys were intended to support the clinicians in the team with implementing the videos into routine practice. The evaluation was overseen by two senior academics who did not have direct involvement with the development of the videos.

One limitation is that we did not collect demographic data on the families so cannot tell the socioeconomic, health literacy or ethnicity of families. It is impossible to know how selection bias affected the results: We may have recruited families who are more engaged in their child's care or families who felt unprepared and sought help through Facebook groups and charities. Further research would be needed to assess the extent of families' learning from these videos (e.g., improvements in a test of knowledge), and whether there are any cost savings of the videos for NHS services (e.g., reduction in callouts to community teams for advice and assistance).

## 5 CONCLUSIONS

The library of videos was perceived as acceptable and valuable to both family carers and healthcare professionals. They form a critical part of a training and support package, supporting families at different time points in their learning journey. The videos were intentionally designed to feature families and a range of different healthcare professionals and to provide emotional support to families as well as practical advice. A key advantage of videos over verbal information from healthcare professionals is that videos can be watched as a refresher as needed and serve as a source of support which can be accessed 24‐7.

Developing the videos has been a real collaboration between researchers, families and healthcare professionals from the hospital and community. Healthcare organizations need to work with families and clinicians from across different services to codesign family‐centred resources to support families who provide other types of care at home (e.g., nasogastric tube feeding, stoma care and tracheostomy care). Children and adults with serious chronic conditions are living longer, and more of the burden of care is placed on families. The importance of high‐quality training and support for families who perform medical procedures for their loved ones will only become more critical as time goes on.

## CONFLICT OF INTERESTS

The authors declare that there are no conflict of interests.

## ETHICS STATEMENT

The evaluation of the videos was approved as a service evaluation by Oxford University Hospitals NHS Foundation Trust and the University of Oxford.

## AUTHOR CONTRIBUTIONS

All authors were part of the stakeholder group involved in developing the videos. Tania Beale was a parent representative in the group. Clinicians Alex CH Lee, Emily J. Harrop and Alison Sharrard oversaw the content of the videos from a clinical perspective. All authors were involved in the development of the evaluation surveys. Bethan Page analysed the data and drafted the paper with support from Charles A. Vincent and Nick Yeung. All authors commented and contributed to the revisions. All authors read and approved the final version.

## Supporting information

Supporting information.

Supporting information.

## Data Availability

The data that support the findings of this study are available on request from the corresponding author. The data are not publicly available due to privacy or ethical restrictions.
