# Supplementary material for: Coproducing a library of videos to support families caring for children with gastrostomies: A mixed‐methods evaluation with family carers and clinicians
Source: Health Expect. 2022 Feb 9;25(3):1038–47. doi: 10.1111/hex.13449 (PMC9122434; doi:10.1111/hex.13449)
Supplement: Supplementary file 1 — Supporting information. [file HEX-25--s001.docx]

SUPPLEMENTARY FILE 1

Full surveys: Family carer and healthcare professional versions

Evaluation of videos on gastrostomy care by families (Family version of survey)

Survey Flow

Standard: About the project (5 Questions)

Standard: You and your child (4 Questions)

Standard: The videos (17 Questions)

Block: Content of videos (6 Questions)

Standard: Usage of videos in practice (5 Questions)

Standard: Final questions (4 Questions)

Standard: Thank You (4 Questions)

| Page Break |  |
| --- | --- |

Start of Block: About the project

Q1.1 **Gastrostomy care: Evaluation of videos**

**About the survey**

This survey is for family carers (e.g. mums, dads, grandparents) who care for a child or young person who has a gastrostomy button (e.g. MINI or MIC-KEY), or have a child on the waiting list for surgery for a gastrostomy button.
  
We have created a library of videos and would like to get your feedback.

This survey is part of a longer term project to develop resources to better prepare and support families who are new to gastrostomy care. The project is a collaboration between researchers, parents and healthcare professionals.

The findings will be shared widely with parents, charities, healthcare providers and researchers.

Q1.2


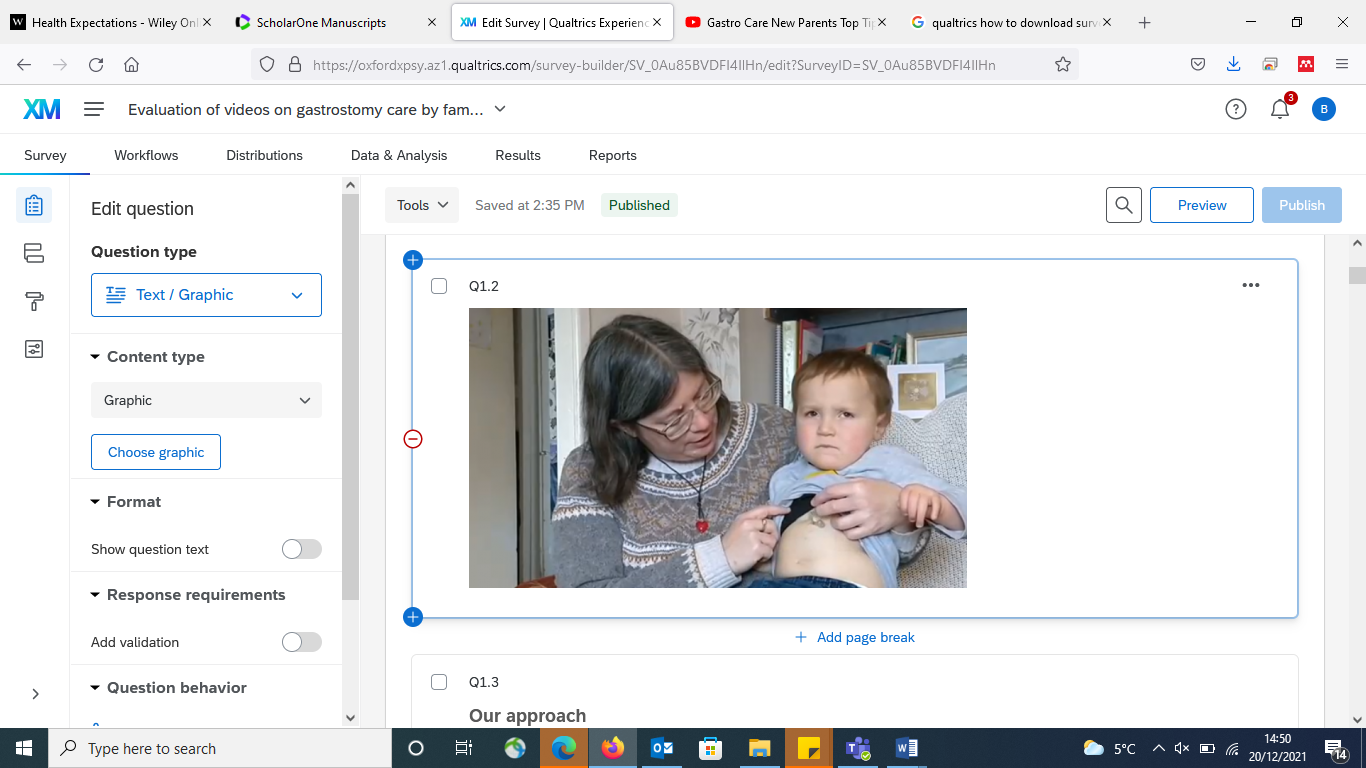


Q1.3
**Our approach**
 
A team of parent representatives, researchers and healthcare professionals from the hospital and community have been involved in creating these videos.


Earlier in the project we did a survey with 150 families to understand their experiences of training and what types of videos would be most helpful. You may have taken part in this.

 
Key things we learnt from the survey:

- Families wanted both healthcare professionals and parents to feature and that they wanted some videos to be filmed at home and to show "real life".
- Families wanted help with managing common problems.
- Families valued advice and tips from more experienced families.

The videos have been created with all this in mind and are primarily aimed at parents. However they may also be valuable for staff training.
 
**A note on consistency of advice from different healthcare professionals**
 
Based on feedback from our team, we believe the information in the videos is consistent with safe and good practice in our region. We are aware there are minor differences in guidelines across the country, with parents often receiving slightly different information and advice from different professionals. What is seen as 'best practice' changes over time and varies between different organisations and different professionals. The videos show a "safe" way of doing procedures, but we have tried to flag in the videos that parents may be told to do things in a slightly different way by the professionals that support them.

Q1.4 **What do I have to do to take part?**

- You will be asked to watched 6 short videos which should take you 15 minutes to do.
- You will then be asked to answer some questions on the videos which will take around 10 minutes to complete. The questions ask you to rate the content of the videos, and how we can make best use of the videos.
- To take part, you need to care for a child under 18 years who has a gastrostomy button (or is on the waiting list for gastrostomy surgery), and you need to be at least 18 years old.
- You will receive a £10 Amazon voucher as a thank you for completing the full survey.
   
  **What will happen with your data?**
- The project has been approved as a Service Evaluation by the University of Oxford and Oxford University Hospitals NHS Foundation Trust. All data will be anonymous. You will not be asked to give any personal data. The data may be reported in academic papers, a PhD thesis and at conferences/presentations.

Q1.5 **THANK YOU FOR YOUR TIME**

End of Block: About the project

Start of Block: You and your child

Q2.1
**About you and your child**
 
*Section 1 of 5:*
  *In this first section we would like to ask some questions about you and the child or young person you provide care for.*

Q2.2 What is your relationship to the child that you care for?

- Mother (1)
- Father (2)
- Other [please specify] (4) ________________________________________________

Q2.3 How old is your child?

________________________________________________________________

Q2.4 How long ago did your child have surgery for their gastrostomy button?

- My child is on the waiting list for surgery (5)
- Less than a year (1)
- 1 - 2 years (2)
- 3 - 4 years (3)
- 5 + years (4)

End of Block: You and your child

Start of Block: The videos

Q3.1
**The videos**
 
*Section 2 of 5: In this section you will be asked to watch some of the videos we have created.

 Below is a list of topics and some example videos for you watch now. There will be a link at the end of the survey to watch the whole set of videos if you want to.*

Q3.2


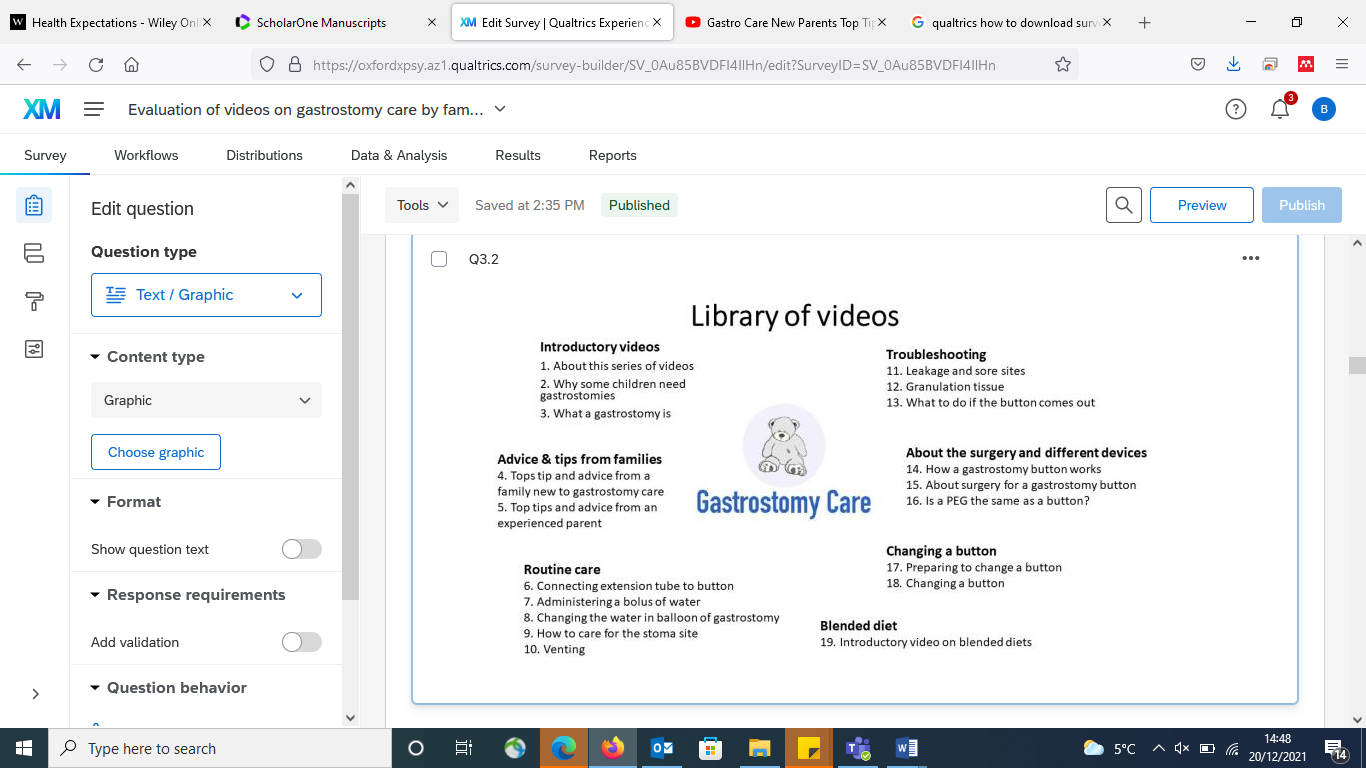


Q3.3
**List of videos**
 
**There are 19 videos in total. Below is the full list of topics. Please read through the list carefully.**
 
**On the next page, you will be asked to watch 6 example videos. These six videos are in highlighted in red in the list.**
 
**Introductory videos**
1. About this series of videos
2. Why do some children need gastrostomies? *
3. What is a gastrostomy?
 
**Advice and tips from families**
4. Advice and tips from a family new to gastrostomy care *
5. Advice and tips from an experienced family
 
**Routine care**
6. Connecting extension tube to button
7. Administering a bolus of water
8. Changing the water in balloon of gastrostomy *
9. How to care for the stoma site
10. Venting
 
**Troubleshooting**
11. Leakage and sore sites
12. Granulation tissue *
13. What to do if the button comes out *
 
**About the surgery and different devices**
14. How a gastrostomy button works
15. About surgery for a gastrostomy button *
16. Is a PEG the same as a button?
 
**Changing a button**

17. Preparing to change a button
18. Changing a button
 
**Blended diet**
19. Introductory video on blended diets
 
 

| Page Break |  |
| --- | --- |

Q3.4 **Please watch all 6 example videos below. You will be then asked some questions about the videos. There is a box for brief comments after each video.**

Q3.5 : <https://youtu.be/mefNF5GyOi0> [Why do some children need gastrostomies]

Q3.6 Do you have any comments on this video?

________________________________________________________________

Q3.7 : <https://youtu.be/5nPmgslu6II> [Top tips from a new parent]

Q3.8 Do you have any comments on this video?

________________________________________________________________

Q3.9 : <https://youtu.be/bU7Ty41EpR8> [Changing the water in the balloon of a gastrostomy button]

Q3.10 Do you have any comments on this video?

________________________________________________________________

Q3.11 <https://youtu.be/f4LCdYabCJ0> [About the surgery for a gastrostomy button]

Q3.12 Do you have any comments on this video?

________________________________________________________________

Q3.13 <https://youtu.be/GH23JRZo0IQ> [What to do if the button falls out]

Q3.14 Do you have any comments on this video?

________________________________________________________________

Q3.15 <https://youtu.be/YsA4k4n665Y> [Granulation tissue]

Q3.16 Do you have any comments on this video?

________________________________________________________________

End of Block: The videos

Start of Block: Content of videos

| Q4.1  **Evaluation of content of videos**     *Section 3 of 5:*      *We would like to get your feedback on the content of the videos.*   *Please rate each statement from 'strongly agree' to 'strongly disagree'.* | Strongly disagree (6) | Somewhat disagree (7) | Neither agree nor disagree (8) | Somewhat agree (9) | Strongly agree (10) |
| --- | --- | --- | --- | --- | --- |
| The information in the videos is easy to understand (1) |  |  |  |  |  |
| There is a good mixture of healthcare professionals and families in the videos (2) |  |  |  |  |  |
| I have no concerns about the accuracy of advice given in the videos (3) |  |  |  |  |  |
| The videos are an appropriate length (4) |  |  |  |  |  |
| There is a good range of topics covered (9) |  |  |  |  |  |
| The videos will help prepare parents to care for their child's gastrostomy at home (5) |  |  |  |  |  |
| The videos will help families to feel more confident (7) |  |  |  |  |  |
| The videos will be useful to families new to gastrostomy care (6) |  |  |  |  |  |
| The videos will be useful to families who are more experienced at caring for their child's gastrostomy (11) |  |  |  |  |  |
| I would recommend the videos to other parents (10) |  |  |  |  |  |

Q4.2 What do you like most about the videos?

________________________________________________________________

________________________________________________________________

________________________________________________________________

________________________________________________________________

________________________________________________________________

Q4.3 Which videos did you find most helpful and why?

________________________________________________________________

________________________________________________________________

________________________________________________________________

________________________________________________________________

________________________________________________________________

Q4.4 How could the videos be improved?

________________________________________________________________

________________________________________________________________

________________________________________________________________

________________________________________________________________

________________________________________________________________

Q4.5 Did you learn anything new from the videos?

- No (1)
- Yes, a little (2)
- Yes, a lot (3)

Q4.6 Please briefly describe what you learnt from the videos

________________________________________________________________

________________________________________________________________

________________________________________________________________

________________________________________________________________

________________________________________________________________

End of Block: Content of videos

Start of Block: Usage of videos in practice

Q5.1 **Using the videos in practice**
 
*Section 4 of 5: In the last section we would like to understand how families  want to use and access the videos.*

Q5.2
**We want to understand which videos would be most helpful for families to watch at what time. When would you want to watch these videos? You can select more than one option.**
**The picture above shows you which videos are included for each topic.**


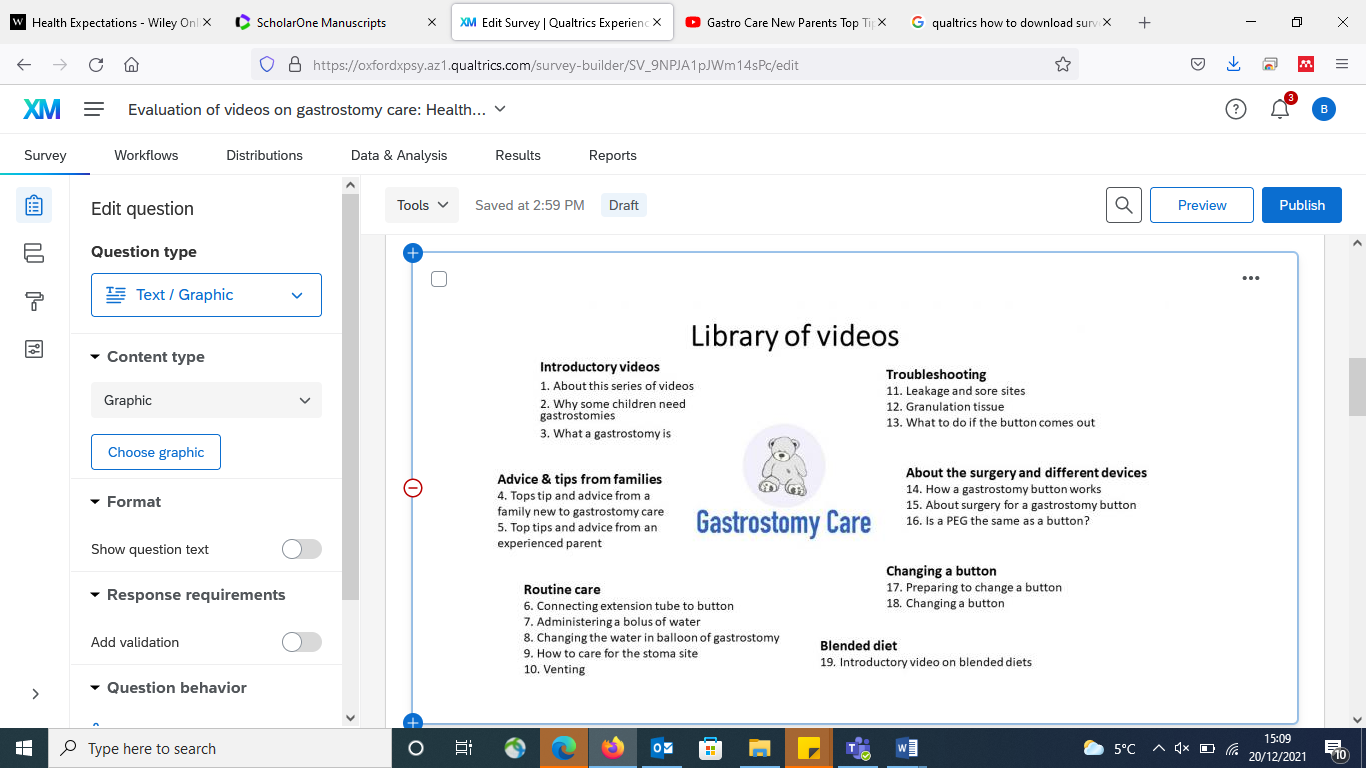


|  | When referred to hospital team for a gastrostomy (1) | Around the time of surgery (2) | In the first few weeks at home after surgery (3) | After my child has had their gastrostomy for a few months or years (5) | Not sure (6) |
| --- | --- | --- | --- | --- | --- |
| Introductory videos (1) |  |  |  |  |  |
| Advice & tips from other parents (7) |  |  |  |  |  |
| Routine care (e.g. how to clean the site, changing water in the balloon) (2) |  |  |  |  |  |
| Troubleshooting (e.g. overgranulation, leakage) (3) |  |  |  |  |  |
| Changing a button (4) |  |  |  |  |  |
| About the surgery and different devices (5) |  |  |  |  |  |
| Blended Diet (9) |  |  |  |  |  |

Q5.3 Do you have any other comments on which videos you would want to watch when?

________________________________________________________________

________________________________________________________________

________________________________________________________________

________________________________________________________________

________________________________________________________________

Q5.4 Are there any dangers or risks of watching any of these videos?

________________________________________________________________

________________________________________________________________

________________________________________________________________

________________________________________________________________

________________________________________________________________

End of Block: Usage of videos in practice

Start of Block: Final questions

Q56
**FINAL REFLECTIONS**
 
*Section 5 of 5: This last section is for any final reflections*

Q6.1 Are there any other topics you would like to see covered in the videos?

________________________________________________________________

________________________________________________________________

________________________________________________________________

________________________________________________________________

________________________________________________________________

Q6.2 Are there any organisations you know of who we should contact to make them aware of these videos?

________________________________________________________________

Q6.3 Do you have any final comments for us on the videos?

________________________________________________________________

________________________________________________________________

________________________________________________________________

________________________________________________________________

________________________________________________________________

End of Block: Final questions

Start of Block: Thank You

Q6.1 Thank you very much for your time today. Your thoughts and advice will be really helpful to us as we develop resources for preparing and supporting families.

Q6.2 You can watch the rest of the videos by clicking on this link: <https://www.oxstar.ox.ac.uk/more/supporting-parents/videos>

Q6.3 As a thank you for your help we will be sending participants who complete the full survey a voucher (£10 Amazon e-giftcard). Please leave your email address here so we can send it to you (we will aim to email it to you within two weeks).

________________________________________________________________

Q6.4 Would you like to be emailed a summary of the findings from this project?

- Yes (please enter your email address) (1) ________________________________________________
- No (2)

End of Block: Thank You

Evaluation of videos on gastrostomy care: Healthcare Professionals

Start of Block: About the project

Q1.1   **Gastrostomy care: Evaluation of videos**

**About this survey**

This survey is for healthcare professionals who care for children and young people who have gastrostomies (e.g. community nurses, specialist surgical nurses & hospital-based children's nurses, paediatricians, surgeons/surgical registrars, respite and school staff, dieticians)

We have created a library of videos featuring parents, children's nurses, a surgeon and surgical nurse, and paediatricians. We have developed the videos based on feedback from families. The videos are mostly about care of a gastrostomy button (MINI or MIC-KEY), but also refer to other devices such as PEGs.

The aim of the survey is to get your feedback on the videos and how we can make best use of them.

This survey is part of a project to develop resources to better prepare and support families who are new to gastrostomy care. The project is a collaboration between researchers, parents and healthcare professionals. The findings will be shared widely with parents, charities, healthcare providers and researchers.

Q1.2
**Our approach**
 
A multidisciplinary team of healthcare professionals from the hospital and community have been involved in developing these videos, as well as parent representatives and researchers.
 
Earlier in the project we conducted a survey with 150 families to understand their experiences of training and what types of videos would be most helpful.
 
Families told us:

- They wanted both healthcare professionals and parents to feature and that they wanted some videos to be filmed at home and to show "real life".
- They wanted help with managing common problems.
- They valued advice and tips from more experienced families.

The videos have been created with all this in mind and are primarily aimed at parents. However they may also be valuable for staff training.
 
**A note on consistency of clinical practice**
 
Based on feedback from our team, we believe the information in the videos is consistent with safe and good practice in our region. We are aware there are minor differences in guidelines across the country, with parents often receiving slightly different information and advice from different professionals. What is seen as 'best practice' changes over time and varies between different organisations and different professionals. The videos show a "safe" way of doing procedures, but we have tried to flag in the videos that parents may be told to do things in a slightly different way by the professionals that support them.

Q1.3   **What do I have to do to take part?**

- You will be shown a series of videos we have created. These will take you about 15 minutes to watch.
- You will then be asked to answer some survey questions on the videos which will take around 10 minutes to complete. The questions ask you to rate the content of the videos, and how we can make best use of the videos.
- To take part, you need to be a healthcare professional who supports children with gastrostomies as part of your role.
- The project has been approved as a service evaluation by the University of X and X NHS Foundation Trust.
- You will be not be asked for any personal data. Your data will be anonymous.

Q1.4 **THANK YOU FOR YOUR TIME**

End of Block: About the project

Start of Block: Your role

Q2.1
**About you**
 
*Section 1 of 5:*
  *In this first section we would like to ask some questions about your role*

Q2.2 Which of the following best describes your job role?

- Community Children's Nurse (CCN) (8)
- Hospital nurse (e.g. specialist surgical nurse, children's ward nurse) (10)
- Surgeon or surgical registrar (9)
- Paediatrician (12)
- Other community healthcare professionals (e.g. respite service/hospice, school) (11)
- Dietician (14)
- Other [please specify] (13) ________________________________________________

Q2.3 Please give your exact job title

________________________________________________________________

Q2.4 During your career have you been involved in teaching parents to care for a gastrostomy?

- Yes (1)
- No (2)

Q2.5 During your career have you been involved in teaching staff to care for a gastrostomy?

- Yes (1)
- No (2)

End of Block: Your role

Start of Block: The videos

Q3.1
**The videos**
 
*Section 2 of 5: In this section you will be asked to watch some of the videos we have created.

 Below is a list of topics and some example videos.*

Q3.2


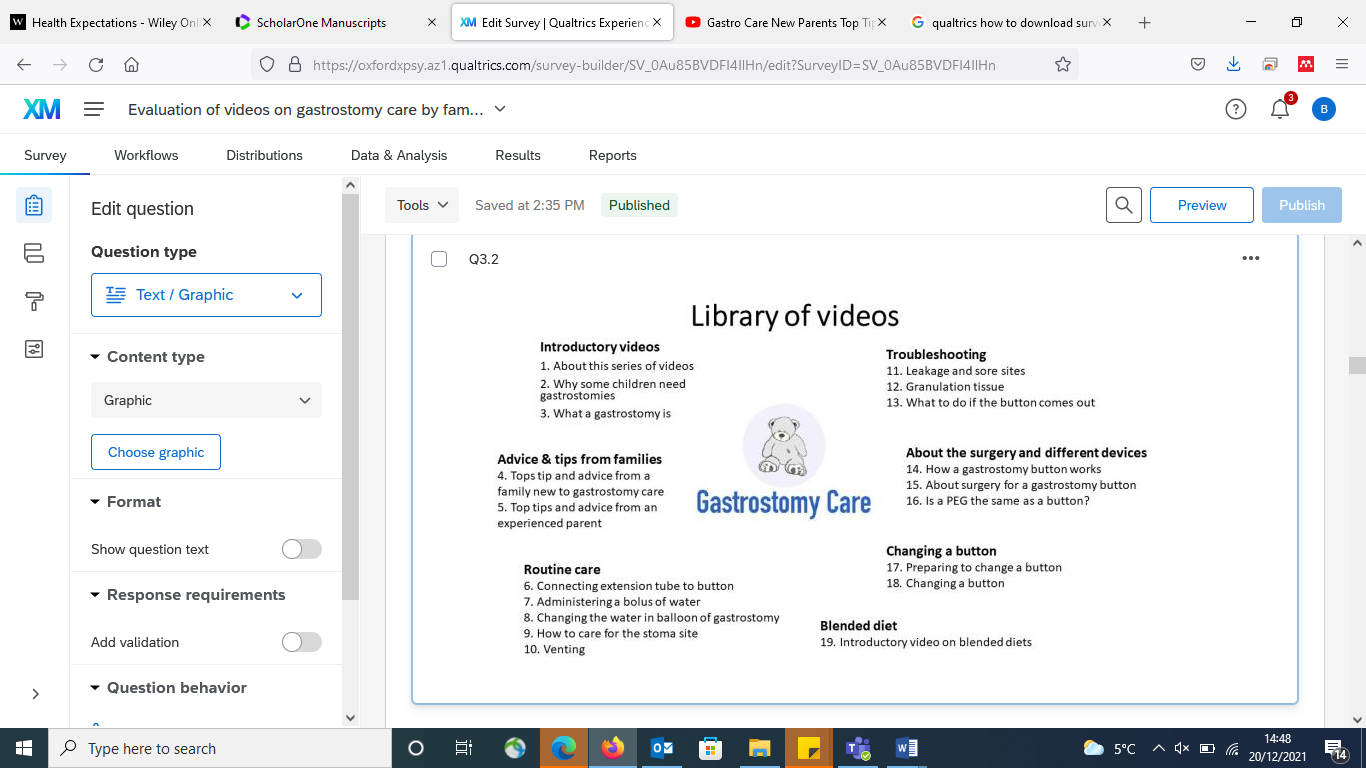


Q3.3
**List of videos**
 
**There are 19 videos in total. Below is the full list of topics. Please read through the list carefully.**
 
**On the next page, you will be asked to watch 6 example videos. These six videos are highlighted in red in the list below.**
 
**Introductory videos**
1. About this series of videos
2. Why do some children need gastrostomies? *
3. What is a gastrostomy?
 
**Advice and tips from families**
4. Advice and tips from a family new to gastrostomy care *
5. Advice and tips from an experienced family
 
**Routine care**
6. Connecting extension tube to button
7. Administering a bolus of water
8. Changing the water in balloon of gastrostomy *
9. How to care for the stoma site

10. Venting
 
**Troubleshooting**
11. Leakage and sore sites
12. Granulation tissue *
13. What to do if the button comes out *

 
**About the surgery and different devices**
14. How a gastrostomy button works
15. About surgery for a gastrostomy button *
16. Is a PEG the same as a button?
 
**Changing a button**

17. Preparing to change a button
18. Changing a button
 
**Blended diet**
19. Introductory video on blended diets
 

Q3.5 **PLEASE WATCH THESE EXAMPLE VIDEOS CAREFULLY**

Q3.5 : <https://youtu.be/mefNF5GyOi0> [Why do some children need gastrostomies]

Q3.6 Do you have any comments on this video?

________________________________________________________________

Q3.7 : <https://youtu.be/5nPmgslu6II> [Top tips from a new parent]

Q3.8 Do you have any comments on this video?

________________________________________________________________

Q3.9 : <https://youtu.be/bU7Ty41EpR8> [Changing the water in the balloon of a gastrostomy button]

Q3.10 Do you have any comments on this video?

________________________________________________________________

Q3.11 <https://youtu.be/f4LCdYabCJ0> [About the surgery for a gastrostomy button]

Q3.12 Do you have any comments on this video?

________________________________________________________________

Q3.13 <https://youtu.be/GH23JRZo0IQ> [What to do if the button falls out]

Q3.14 Do you have any comments on this video?

________________________________________________________________

Q3.15 <https://youtu.be/YsA4k4n665Y> [Granulation tissue]

Q3.16 Do you have any comments on this video?

________________________________________________________________

End of Block: The videos

Start of Block: Content of videos

Q4.1
**Evaluation of content of videos**

*Section 3 of 5:*  *We would like to get your feedback on the content of the videos.*
*Please rate each statement from 'strongly disagree' to 'strongly agree'.*

|  | Strongly disagree (6) | Somewhat disagree (7) | Neither agree nor disagree (8) | Somewhat agree (9) | Strongly agree (10) |
| --- | --- | --- | --- | --- | --- |
| The information in the videos is easy to understand (1) |  |  |  |  |  |
| There is a good mixture of healthcare professionals and families in the videos (2) |  |  |  |  |  |
| The information in the videos is consistent with best practice guidance (3) |  |  |  |  |  |
| The videos are an appropriate length (4) |  |  |  |  |  |
| There is a good range of topics covered (9) |  |  |  |  |  |
| The videos will help prepare parents to care for their child's gastrostomy at home (5) |  |  |  |  |  |
| The videos will help families to feel more confident (11) |  |  |  |  |  |
| The videos will be useful to families new to gastrostomy care (6) |  |  |  |  |  |
| The videos will be useful to families who are more experienced at caring for their childs gastrostomy (7) |  |  |  |  |  |
| I would recommend these videos to parents (12) |  |  |  |  |  |
| I would recommend these videos to my colleagues (10) |  |  |  |  |  |

Q4.2 What do you like most about the videos?

________________________________________________________________

________________________________________________________________

________________________________________________________________

________________________________________________________________

________________________________________________________________

Q4.3 How could the videos be improved?

________________________________________________________________

________________________________________________________________

________________________________________________________________

________________________________________________________________

________________________________________________________________

Q4.4 Have the videos aided your own learning?

- No (1)
- Yes, a little (2)
- Yes, a lot (3)

Q4.5 Please briefly describe anything you have learnt from the videos

________________________________________________________________

________________________________________________________________

________________________________________________________________

________________________________________________________________

________________________________________________________________

End of Block: Content of videos

Start of Block: Usage of videos in practice

Q5.1 **Using the videos in practice**
 
*Section 4 of 5: In this section we would like to understand how to make best use of the videos.*

Q5.2
**We want to understand which videos would be most helpful for families to watch at what time. When do you think families should watch these videos? You can select more than one option.**

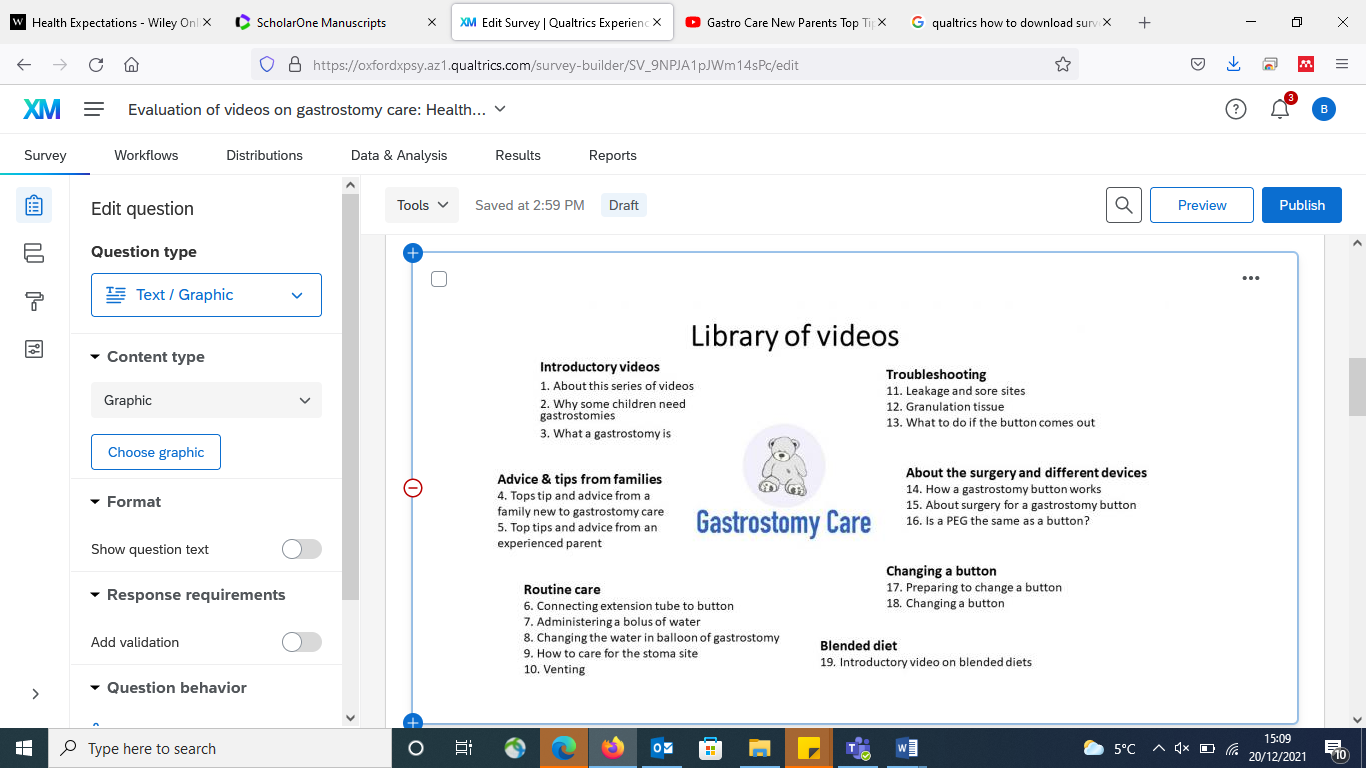


**The picture above shows you which videos are included for each topic.**

|  | When referred to hospital team for a gastrostomy (1) | Around the time of surgery (2) | In the first few weeks at home after surgery (3) | After child has had their gastrostomy for a few months or years (5) | Not sure (6) |
| --- | --- | --- | --- | --- | --- |
| Introductory videos (1) |  |  |  |  |  |
| Advice & tips from other parents (7) |  |  |  |  |  |
| Routine care (e.g. how to clean the site, changing water in the balloon) (2) |  |  |  |  |  |
| Troubleshooting (e.g. overgranulation, leakage) (3) |  |  |  |  |  |
| Changing a button (4) |  |  |  |  |  |
| About the surgery and different devices (5) |  |  |  |  |  |
| Blended Diet (9) |  |  |  |  |  |

Q5.3 Do you have any other comments on which videos parents should watch when?

________________________________________________________________

________________________________________________________________

________________________________________________________________

________________________________________________________________

________________________________________________________________

Q5.4 Are there any dangers or risks of parents watching any of these videos?

________________________________________________________________

________________________________________________________________

________________________________________________________________

________________________________________________________________

________________________________________________________________

Q5.5 How might you make use of these videos in your practice?

________________________________________________________________

________________________________________________________________

________________________________________________________________

________________________________________________________________

________________________________________________________________

Q5.6 Would these videos be useful for staff training? If yes, which staff groups would benefit?

________________________________________________________________

________________________________________________________________

________________________________________________________________

________________________________________________________________

________________________________________________________________

End of Block: Usage of videos in practice

Start of Block: Final reflections

Q5.1
**FINAL REFLECTIONS**
 
*Section 5 of 5: This last section is for any final reflections*

Q5.2 Are there any other topics you would like to see covered in the videos?

________________________________________________________________

________________________________________________________________

________________________________________________________________

________________________________________________________________

________________________________________________________________

Q5.3 Are there any organisations you know of who we should contact to make them aware of these videos?

________________________________________________________________

Q5.4 Do you have any final comments for us on the videos?

________________________________________________________________

________________________________________________________________

________________________________________________________________

________________________________________________________________

________________________________________________________________

End of Block: Final reflections

Start of Block: Thank You

Q6.1 Thank you very much for your time today. Your thoughts and advice will be really helpful to us as we develop resources for preparing and supporting families.

Q63 **You can watch the rest of the videos by clicking on this link:** <https://www.oxstar.ox.ac.uk/more/supporting-parents/videos>

Q6.2 As a thank you for your help we will be sending participants who complete the full survey a voucher (£10 Amazon e-giftcard). Please leave your email address here so we can send it to you (we will aim to email it to you within two weeks).

________________________________________________________________

Q6.3 Would you like to be emailed a summary of the findings from this project?

- Yes [please provide your email address] (4) ________________________________________________
- No (5)

End of Block: Thank You
