# Supplementary material for: Coproducing a library of videos to support families caring for children with gastrostomies: A mixed‐methods evaluation with family carers and clinicians
Source: Health Expect. 2022 Feb 9;25(3):1038–47. doi: 10.1111/hex.13449 (PMC9122434; doi:10.1111/hex.13449)
Supplement: Supplementary file 2 — Supporting information. [file HEX-25--s002.docx]

**Supplementary File 2: Summary of families and healthcare professionals’ comments on individual videos**

Video 1: Why children need gastrostomies

Most of the comments on this video related the short-length or clarity of the video, e.g. “*good clear explanation is provided and in simple terms so that anyone that is not medically minded can understand [family carer]*”. There were a few suggestions for extra details, e.g. other less common reasons why children might need gastrostomies, or improvements to the production quality.

Video 2: Advice and tips from a new parent

Healthcare professionals and families found the video reassuring and informative and valued hearing directly about a family’s experience and tips. One family carer commented: “*I found this video really moving. It was helpful and reassuring to hear about the emotions involved for this family. It made the whole process seem a bit less daunting and more real*.” As some family carers remarked, the family’s story in the video is not typical of all families: “*Helpful but not relevant to all parents as some children that have complex medical needs would have a very different path for their child*.” A few participant suggested that it could be helpful to film more videos with different families talking about their experiences to increase the diversity of families involved.

Video 3: About the surgery for a gastrostomy button

Most family carers and healthcare professionals commented that the video was clear and informative. Several healthcare professionals commented on the clear definitions: “*Glad to see that complex words are explained. Keeping language simple/layman's terms is so important in these types of videos.*” Some family carers commented that they had learnt new things from the video, although their child had their surgery a long time ago: “*This video is REALLY interesting in how the stomach is attached to the tummy wall - our child had issues in this area on one operation so actually really interesting to watch.”* A few families commented that this was not how all gastrostomy surgery works, and that a different surgical technique was used for their child.

Video 4: Changing the water in a gastrostomy balloon

Several healthcare professionals commented that they liked that a parent was doing the teaching in the video, demonstrating on her child at home, with additional diagrams and voiceovers added: “*This is a really lovely combination of the 'real life' setting, with the voice over and the diagrams and text*.” There were several comments about the fact sterile water was used in the videos: participants commented that some areas suggest cooled boiled water and some families use tap water. A few families commented on small differences in practice between how they were taught and what the mother in the video does; however these small concerns were not generally raised in the comments by healthcare professionals. For example, a few family carers were concerned the procedure was done with the child on the mother’s lap rather than lying on a bed; although the voiceover specifies that it is best when you are learning to do the procedure on a bed, some parents still had concerns. Conversely a few commented that it shows how easy and relaxed the procedure can be: “*I love that this video is done with the child on your lap - it really does show this is an easy procedure and nothing to be scared of.”*

Video 5: What to do if the button comes out

Several family carers and healthcare professionals commented that this video was clear and reassuring: “*Another really informative video. We currently have NG tube which is always coming out. This has reassured us about the process for if/when the button comes out.”* A few healthcare professionals and family carers commented that they had not previously heard of, or managed to get hold of an ‘enplug’ device for if the gastrostomy button comes out. There were also a few suggestions about improving the production quality.

Video 6: Granulation tissue

Family carers and professionals alike commented that the video was clear and provided a useful guide to possible treatments. Several participants commented that they found the photos particularly useful. Healthcare professionals particularly valued this video: “*I found this video extremely useful as this is not an uncommon problem and often causes a lot of frustrations amongst all involved.”* A few healthcare professionals commented that granulation tissue was often something that causes parents to worry, and that this video made help ease some anxieties: “*Good to show this as parents can be concerned about this as it looks like something is coming out and it bleeds easily sometimes leading parents to think there is something seriously wrong.”*
